# Women in Medicine

**Published:** 1921-08-13

**Authors:** 


### Women in Medicine.

THE annual report of the Council of the London (Royal Free Hospital) School of Medicine for Women states that 112 students entered the School during the session 1919 to 1920, making a total of 510 students in attendance. In this number 478 were registered internal students of the University of London. During the session seventeen students took the M.B., B.S. degree of London University, and twenty-three the qualifying diplomas of the Royal Colleges of England. Two former students took the M.D. degree and two the M.S. degree of the University of London. Seven passed the primary Fellowship examination, and two former students obtained the Fellowship of the Royal College of Surgeons of England.

With regard to the scheme for the extension of the School of the Royal Free Hospital, it is announced that the appeal has reached £33,000 in money or promises, and that many activities are in progress to augment this sum. As stated in a previous report, the land for the extension of the hospital has been given, and so soon as sufficient money is in hand and labour conditions have become more normal the foundations of the extension will be commenced.

